# Use of the lignocellulose-degrading bacterium *Caldicellulosiruptor bescii* to assess recalcitrance and conversion of wild-type and transgenic poplar

**DOI:** 10.1186/s13068-020-01675-2

**Published:** 2020-03-11

**Authors:** Christopher T. Straub, Ryan G. Bing, Jack P. Wang, Vincent L. Chiang, Michael W. W. Adams, Robert M. Kelly

**Affiliations:** 1 Department of Chemical and Biomolecular Engineering, North Carolina State University, EB-1, 911 Partners Way, Raleigh, NC 27695-7905 USA; 2 Department of Forestry and Environmental Resources, North Carolina State University, Raleigh, NC 27695 USA; 3 Department of Biochemistry and Molecular Biology, University of Georgia, Athens, GA 30602 USA

**Keywords:** *Caldicellulosiruptor*, Extreme thermophiles, Lignocellulose, Biofuel, Poplar

## Abstract

**Background:**

Biological conversion of lignocellulosic biomass is significantly hindered by feedstock recalcitrance, which is typically assessed through an enzymatic digestion assay, often preceded by a thermal and/or chemical pretreatment. Here, we assay 17 lines of unpretreated transgenic black cottonwood (*Populus trichocarpa*) utilizing a lignocellulose-degrading, metabolically engineered bacterium, *Caldicellulosiruptor bescii*. The poplar lines were assessed by incubation with an engineered *C. bescii* strain that solubilized and converted the hexose and pentose carbohydrates to ethanol and acetate. The resulting fermentation titer and biomass solubilization were then utilized as a measure of biomass recalcitrance and compared to data previously reported on the transgenic poplar samples.

**Results:**

Of the 17 transgenic poplar lines examined with *C. bescii*, a wide variation in solubilization and fermentation titer was observed. While the wild type poplar control demonstrated relatively high recalcitrance with a total solubilization of only 20% and a fermentation titer of 7.3 mM, the transgenic lines resulted in solubilization ranging from 15 to 79% and fermentation titers from 6.8 to 29.6 mM. Additionally, a strong inverse correlation (*R*^2^ = 0.8) between conversion efficiency and lignin content was observed with lower lignin samples more easily converted and solubilized by *C. bescii*.

**Conclusions:**

Feedstock recalcitrance can be significantly reduced with transgenic plants, but finding the correct modification may require a large sample set to identify the most advantageous genetic modifications for the feedstock. Utilizing *C. bescii* as a screening assay for recalcitrance, poplar lines with down-regulation of coumarate 3-hydroxylase 3 (C3H3) resulted in the highest degrees of solubilization and conversion by *C. bescii*. One such line, with a growth phenotype similar to the wild-type, generated more than three times the fermentation products of the wild-type poplar control, suggesting that excellent digestibility can be achieved without compromising fitness of the tree.

## Background

Lignocellulosic biomass contains three of the most abundant polymers on Earth—cellulose, hemicelluloses, and lignin—generated via solar-powered fixation of carbon dioxide. This abundant resource is available from crop wastes, dedicated cultivated feedstocks, and sustainable harvest of forest lands, to name a few sources [10]. In addition, more than half of land-based biomass is carbohydrate in the form of biopolymers, primarily cellulose, a relatively homogenous linear polymer of β-1,4-linked glucose. In angiosperms, the other major carbohydrate components, hemicelluloses, are heterogeneous polymers of primarily xylose along with smaller amounts of arabinose, mannose, rhamnose, galactose, glucose and glucuronic acid [9].

While these substrates are rich in carbohydrate content, the barrier to biomass conversion of the carbohydrate content is the recalcitrance of renewable feedstocks [6], which has been shown to be a strong function of lignin content [16]. In attempts to reduce recalcitrance, transgenic trees and grasses have been generated through a variety of molecular strategies [24], although the efficacy of microbial conversion of these biomasses to fermentation products is highly variable [18, 19] and significantly dependent on pretreatment conditions. Such transgenic modifications have ancillary consequences, such as effects on growth. Thus, striking a balance between reducing feedstock recalcitrance, often by lowering lignin content, and achieving excellent growth and fitness under field conditions is a key challenge for developing renewable transgenic biomasses.

In order to determine the suitability of transgenic feedstocks for production of bio-based chemicals, direct deconstruction by microorganisms can provide insight that complements evaluations based on standard simultaneous saccharification and fermentation (SSF) assays utilizing exogenously added enzyme cocktails. *Caldicellulosiruptor bescii* is an extremely thermophilic bacterium, capable of degrading unpretreated lignocellulosic biomass and deconstructing and metabolizing the cellulose and hemicelluloses [2]. Furthermore, *C. bescii* has been metabolically engineered to produce non-native fermentation products, such as ethanol [3]. In addition to prospects for using *C. bescii* as a platform organism for direct processing of unpretreated biomass, there is also the prospect of using this bacterium to screen for the efficacy of transgenic manipulations of lignocellulosic biomass to reduce recalcitrance. Here, previously generated and reported transgenic poplar lines with a broad variation in the lignin content and composition [28] were subjected to fermentation by *C. bescii* to assess both recalcitrance and convertibility to bio-based ethanol and acetate.

### Monolignol targeted transgenic poplar lines

**Fig. 1:**
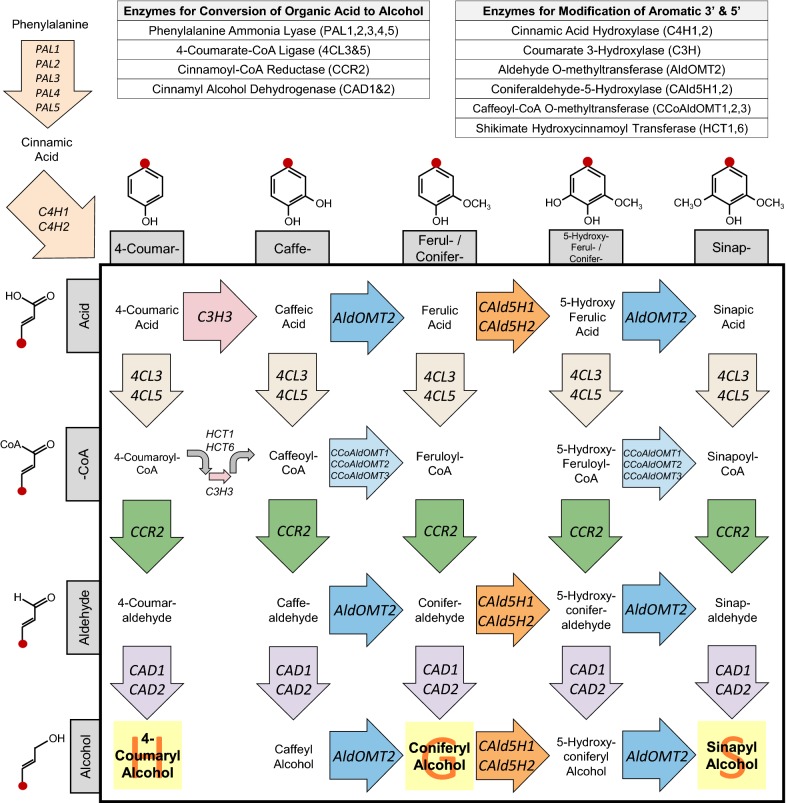
Metabolic pathway grid for monolignol biosynthesis present in angiosperms. Monolignol biosynthesis from phenylalanine with enzymes responsible for conversion of phenylalanine to 4-coumaric acid, modification of the 3′ and 5′ side groups on the aromatic ring, and the conversion of organic acid to alcohol on the three carbon branch at 1′ position on the aromatic ring

Lignin is the primary component of plant cell walls responsible for biomass recalcitrance [13]. Many studies have attempted to not only reduce its content as a fraction of the biomass, but also to modify its composition and linkage structures by down-regulation of genes in the biosynthetic pathways of monolignols or enzymes involved in lignin polymerization [16]. Monolignol biosynthesis can be viewed in terms of three major steps: (1) production of 4-coumaric acid from phenylalanine; (2) modification of aromatic ring side groups to hydroxy or methoxy moieties at positions 3 and 5; and (3) conversion of the three-carbon branch from an organic acid to an alcohol (Fig. 1). Various genetic strategies to modify the monolignol synthesis pathway have been utilized to generate transgenic poplar lines with reduced recalcitrance (for examples of previous efforts, see Table 1). Most have involved strategies that manipulate a single gene in each transgenic line. Improvements in conversion of the carbohydrate content to sugars from these efforts have likewise been highly variable.

**Table 1:** Examples of transgenic poplar lines targeting native monolignol synthesis genes

Populus species	Target gene(s)	Number transgenic lines reported	Lignin (wt%)	*S*/*G* ratio	Carbohydrate content data	Source
*Populus tremuloides Michx.*	4-Coumarate:CoA ligase (4CL1)	8	11.84–20.60 (21.6)	–	Cellulose (wt%) 45.95–50.83 (44.23)	[7]
*Populus grandidentata x alba*	*p*-Coumaryl-CoA 3′-hydroxylase (C3′H)	9	10.51–21.03 (23.78)	1.8– 3.1 (1.8)	Glucose (wt%) 46.03–51.10 (45.69) Xylose (wt%) 17.30–21.12 (17.87)	[4, 20]
*Populus tremuloides*	4-Coumarate-CoA ligase (4CL1)	5	13.1–16.0 (22.2)	2.1–2.3 (2.2)	Cellulose (wt%) 43.1–47.3 (41.4)	[12]
	Coniferaldehyde 5-hydroxylase (CAld5H)	5	19.7–22.4 (22.2)	3.0–5.5 (2.2)	Cellulose (wt%) 40.0–44.7 (41.4)	
	4CL1 Down-regulation and CAld5H Upregulation	4	10.7–13.7 (22.2)	2.6-3.6 (2.2)	Cellulose (wt%) 49.2–53.3 (41.4)	
*Populus tremula x alba*	4-Coumarate:CoA ligase (4CL1)	14	–	1.4–2.2 (1.8)	–	[26]
*Populus tremula x alba*	Cinnamoyl-CoA reductase (CCR)	2^a^	19.1–19.6 (20.7)	1.9 (1.9)	Cellulose (wt%) 51.7–53.8 (50.9) Hemi-cellulose (wt%) 19.7–20.9 (19.7)	[25]
		2^b^	16.6–19.8 (21.7)	1.9–2.1 (2.0)	Cellulose (wt%) 53.6–56.1 (52.4) Hemi-cellulose (wt%) 19.7––20.7 (19.2)	
*Populus tremula x alba*	Caffeic acid/5-hydroxyferulic acid *O*-methyltransferase (COMT)	2^c^	19.3–19.5 (19.0)	1.5 (2.2)	–	[11]
	Cinnamoyl alcohol dehydrogenase (CAD)	3^c^	18.0–18.3 (19.0)	2.0 (2.2)	–	
*Populus tomentosa*	4-Coumarate:CoA ligase (4CL)	2	22.16–22.68 (24.12)	–	Cellulose (wt%) 49.58–51.53 (47.94)	[27]
	Caffeoyl-CoA 3-*O*-methyltransferase (CCoAOMT)	2	21.6–21.8 (24.1)	–	Cellulose (wt%) 50.41–51.75 (47.94)	
*Populus nigra x maximowiczii*	4-Coumarate:CoA ligase (4CL)	3^d^	22.5–23.7 (23.6)	*S*/*V* ratio^e^ 0.6–1.9 (1.8)	Glucan (wt%) 36.1–39.7 (37.5) Xylan (wt%) 13.8–14.9 (14.1)	[30]

Values in parenthesis denote value reported for control/wild-type

^a^Field trial #1 (referred to as Belgian trial—10 month duration)

^b^Field trial #2 (referred to as French trial—20 month duration)

^c^Lapierre et al. reports data for various time periods in greenhouse and field trials. Data shown here for 6-month-old greenhouse grown trees

^d^Xiang et al. reports data for years 2 and 3 from lines grown in a mountain region and coastal region. Data reported here for year 3 trees grown in mountain region

^e^*S*/*V* ratio is syringaldehyde to vanillin ratio

While multiple separate studies have perturbed monolignol synthesis genes in various *Populus* species, a systems biology-based approach to evaluate the aspects of all of the genes involved in monolignol synthesis provides a more substantial perspective and can be subjected to a more robust analysis. Such a strategy to generate transgenic lines with reduced recalcitrance is likely to be more successful at identifying optimal targets given the intrinsic complexity of monolignol biosynthesis [15]. Along these lines, recent work [28] sought the most promising avenues for modifying *P. trichocarpa* lignin structure and content with an eye towards favorable wood characteristics and plant fitness. The down-regulation of 21 genes involved in monolignol biosynthesis, individually and by gene-pairs and gene families, were considered as a basis for a mathematical model that predicted wood traits as a function of transgenic changes. A collection of transgenic lines with a broad set of phenotypes was generated and characterized [28]. Selected characterization data, such as carbohydrate and lignin content, from that study are summarized in Table 2, along with previously reported transcript abundance relative to the wild type of the target gene(s). The goal here was to determine how these genetic and transcriptomic alterations in the transgenic poplar lines affected microbial biomass solubilization and bioproduct formation and point to further favorable outcomes for bio-based chemical production.

**Table 2:** Selected poplar wood properties with *C. bescii* treatment data

Line	Target genes	Transcript^b^ (% of Ctrl)	Lignin^b^ (wt%)	Total carb^b^ (wt%)	Height^b^ (% of Ctrl)	Diameter^b^ (% of Ctrl)	Stem volume^b^ (% of Ctrl)	*C. bescii* solubilization (wt%)	*C. bescii* fermentation products (mM)
Wild type	–	–	21.7	69.9	100%	100%	100%	20.1 (0.37)	7.3 (0.15)
i20-5	C3H3	13	9.9	86.6	62.2	94.6	49.9	79.3 (0.80)	29.6 (0.48)
i20-10	C3H3	17	13.3	80.7	90.8	109.0	94.7	52.0 (0.71)	22.5 (0.18)
i69-4	C3H3	14	11.9	96.5	66.7	101.8	60.8	64.4 (0.38)	28.1 (0.38)
	C4H1	9							
	C4H2	27							
i69-13	C3H3	20	13.6	91.1	71.5	103.6	67.4	51.1 (0.83)	23.3 (0.55)
	C4H1	14							
	C4H2	36							
a10-8	C4H1	23	20.4	66.4	105.7	101.1	55.8	15.9 (0.43)	6.8 (0.15)
a12-10^a^	4CL3	16	17.4	70.1	ND	ND	ND	21.5 (0.74)	11.7 (0.25)
i15-3^a^	4CL3	3	19.1	71.6	ND	ND	ND	28.5 (0.21)	11.8 (0.06)
	4CL5	10							
i33-5	CAD1	5	21.9	65.6	85.1	103.6	81.1	15.0 (0.19)	8.5 (0.19)
i33-5	CAD1	94	21.4	72.0	96.4	109.0	100.8	24.2 (0.62)	9.1 (0.03)
i35-7	CAD1	6	13.8	68.9	46.6	77.5	26.2	78.2 (0.68)	27.4 (0.17)
	CAD2	81							
i24-1	CCoAOMT1	6	19.7	72.0	69.5	75.2	47.9	21.5 (0.96)	8.9 (0.10)
	CCoAOMT2	7							
i21-6^a^	CCoAOMT3	24	18.7	68.9	94.2	95.7	99.7	26.6 (1.1)	9.9 (0.02)
i30-1	AldOMT2	5	16.4	71.4	65.5	82.8	52.9	46.7 (0.38)	18.8 (0.14)
i6-9	PAL1	4	18.4	74.3	78.3	76.6	66.1	30.8 (0.53)	12.7 (0.09)
	PAL2	4							
i8-1	PAL1	31	17.9	70.0	83.7	107.5	126.0	28.5 (1.4)	11.3 (0.29)
	PAL2	62							
	PAL3	38							
	PAL4&5	120							
a4-3	PAL5	21	14.5	74.9	82.2	83.6	80.7	55.7 (0.47)	9.1 (0.11)
i19-4^a^	HCT1	81	17.6	76.7	47.9	60.7	23.9	41.2 (0.08)	15.8 (0.19)
	HCT6	44							

Note that transgenic poplar lines with more than one target gene (and accompanying transcript level) are listed in subsequent rows below the first target gene

Standard deviation for *n* = 3 technical replicate fermentations shown in parenthesis for solubilization and fermentation products

Further data on poplar samples and properties including carbohydrate composition, lignin properties, and enzymatic saccharification data is available in supplementary sections of [28]

^a^Biomass samples in which technical replicate biomass samples were combined to obtain sufficient material quantities for this study. Values for biomass samples were averaged. Refer to Additional file 1: Table S1 for data on each biomass sample and calculation of average

^b^Previously published data from [28]

## Materials and methods

### Biomass preparation

All wild type and transgenic greenhouse grown *Populus trichocarpa* samples were created and prepared as described elsewhere [28]. The untreated stems of 6-month-old trees were stripped of bark and air dried for approximately 72 h. The dried stem segments were milled utilizing a Wiley Mill and sieved to 40/80 mesh. The 40/80 mesh material was water-washed by adding 1.5 g of material to a 50 mL conical centrifuge tube and filling with deionized water. The centrifuge tube was centrifuged and the supernatant discarded. This was repeated twice more and the pelleted material was dried at 50 °C.

### Biomass solubilization

Following the 7-day incubation of *C. bescii* with the washed biomass, the sealed serum bottles were removed from the shaking incubator and allowed to cool to room temperature. The entire 50 mL contents were transferred to a 50 mL conical centrifuge tube and centrifuged, as described above. A portion of the supernatant was sterile-filtered and saved for fermentation product analysis. The remainder of the supernatant was discarded. Each serum bottle was rinsed with deionized water to remove any remaining biomass and this was added to the biomass pellet in the centrifuge tube. The centrifuge tube was filled with water up to 45 mL, shaken to loosen the pellet, and centrifuged again to pellet the biomass. The supernatant was again removed and another wash performed. After the final wash and removal of the supernatant, the pellet was dried at 50 °C and the weight was recorded to calculate biomass solubilization.

### Biomass properties

Biomass properties, such as lignin content, carbohydrate content, growth phenotypes and others reported in Table 2, were determined and reported previously [28]. Microbial solubilization and fermentation were generated as part of this study and analyzed in part with the previously reported biomass properties.

### Microbial growth on biomass

*Caldicellulosiruptor bescii* was cultured at 50 mL in sealed serums bottles on 5 g/L DSMZ671 defined media with the washed biomass as the only substrate, as described previously [22]. Cultures were incubated at 65 °C for 7 days (with shaking at 150 RPM) after which fermentation products were analyzed and biomass solubilization was measured, as described above.

### Analysis of fermentation products

The sterile-filtered supernatant obtained from the culture was utilized for fermentation product analysis. Acetate was quantified utilizing high-performance liquid chromatography (HPLC) with a Waters Model 2489 UV/Vis detector. Ethanol was quantified via gas chromatography utilizing a Shimadzu GC-2014 (Phenomenex ZB-WAXplus column; Part No. 7HK-G013-22). Nitrogen was utilized as the carrier gas and detection via FID.

## Results and discussion

### *C. bescii* fermentation of transgenic lines of *P. trichocarpa*

Based on previous work [28], 17 transgenic samples of *P. trichocarpa* (Additional file 1: Table S1), along with the wild-type control, were fermented without pretreatment with a metabolically engineered strain of *C. bescii* in which the *adhE* gene (bi-functional alcohol dehydrogenase) from *Clostridium thermocellum* was inserted to enable the generation of ethanol, in addition to its natural fermentation products: acetate, H_2_ and CO_2_ [29]. Poplar stems (bark removed) were milled and sieved to 40/80 mesh, water washed and dried, and incubated with *C. bescii* for 7 days at 65 °C. Prior to this study, *C. bescii* had been examined on two lines of transgenic switchgrass with reduced lignin content, resulting in small improvements in biomass solubilization and fermentation [33]. However, the broader sample set available here—with all samples originating from the same parent line—provided an opportunity to examine *C. bescii* efficacy as a function of recalcitrance factors, especially the lignin content of the wood.

**Fig. 2:**
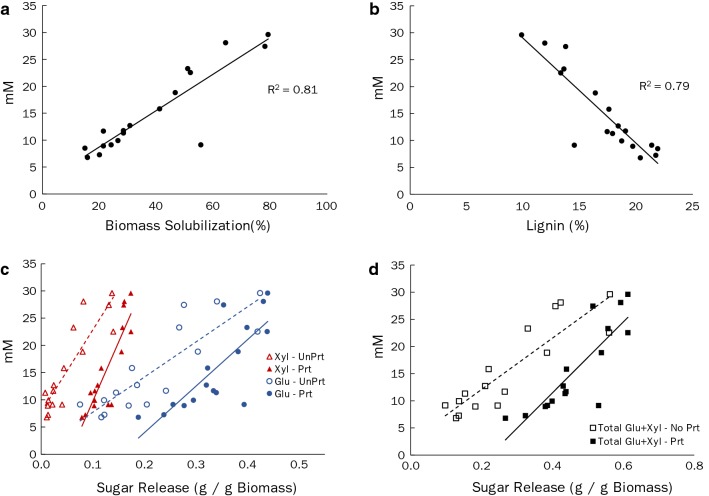
*Caldicellulosiruptor bescii* fermentation production from poplar lines. (**a**) Fermentation production as a function of biomass solubilization after 7-day treatment with *C. bescii*; (**b**) Lignin composition of poplar lines; (**c**) glucose and xylose release from saccharification assay for unpretreated (No Prt) and pretreated (Prt) (5 min in water at 180 °C followed by 72 h enzymatic digestion)

As is shown in Table 2, the transgenic poplar lines that were generated [28] varied significantly in terms of solubilization by *C. bescii* (15 to 79%) and total fermentation products generated (ethanol plus acetate) by *C. bescii* (6.8 to 29.6 mM). While two of the transgenic lines (i20-5 and i35-7) with the highest solubilization and conversion by *C. bescii* have been previously reported [22], this effort aims to extend such analysis to a wider sample set of transgenic poplar lines generated from the same parent line. This includes some lines performing more poorly than the wild-type control (20.1% solubilization and 7.3 mM fermentation products). Overall, fermentation production (mM) by *C. bescii* directly correlated with biomass solubilization (*R*^2^ = 0.81) (Fig. 2a), and inversely with lignin content (*R*^2^ = 0.79) (Fig. 2b). However, there were some unexpected results. One transgenic poplar sample of interest was the a4-3 line (which targeted the down-regulation of PAL5). With a lignin content of 14.5% versus 21.7% for the wild type wood, an expected improvement in solubilization (56%) compared to wild type (20%) was observed. However, the concentration of fermentation products (9.1 mM) was comparable to that of wild type poplar (7.3 mM), even though previously reported enzymatic saccharification levels were substantially above wild type [28]. The reasons for this are unclear. Yet, upon examining the lignin properties previously reported, the a4-3 line has the highest proportion of spirodienone (β-1) interunit linkages (2.9% vs 2.3% for wild type), while the lines targeting C3H3 that performed substantially better than the wild type had 0.0% for lines i20-5, i69-4, and i69-13 and 0.4% for i20-10 [28]. One possibility is that the a4-3 biomass released a compound that was inhibitory to *C. bescii*, suggesting that solubilization could be primarily abiotic. Another possibility is that the carbohydrates remained bound to lignin moieties and, while solubilized, were not available in a form that *C. bescii* could utilize for fermentation.

Another useful comparison for assessment of lignocellulosic substrates is efficacy of the exogenously added enzymatic digestions in comparison to the natively produced degradation enzymes released by *C. bescii*. The release of glucose and xylose from the wood, via enzymatic saccharification (5 min in water at 180 °C followed by 72 h enzymatic digestion), previously reported [28], correlated with *C. bescii* conversion to fermentation products (acetate, ethanol) (Fig. 2c, d). It is important to emphasize that prior enzymatic saccharification assays were performed with wood samples that had been pretreated with acetone to remove extractives, while the wood utilized here for the *C. bescii* treatment was milled without any other form of chemical, thermal, or prior enzymatic pretreatment.

**Fig. 3:**
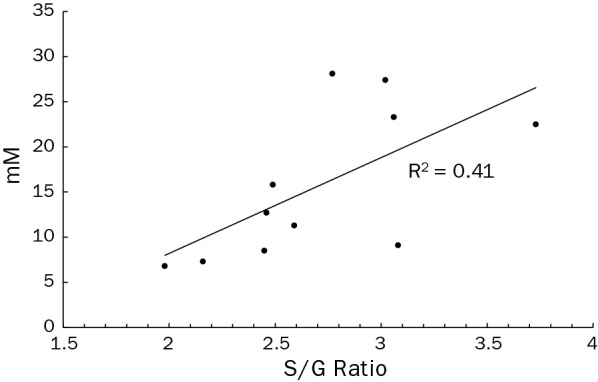
S/G ratio effect on *C. bescii* fermentation products. Syringyl (*S*) over guaiacyl (*G*) monolignol ratio measured in wood samples and its effect on fermentation products. (Line i20-5 data point is (9.9, 29.6) but not charted due to figure scale.)

While the overall lignin content negatively correlated to the yield of fermentation products, the type of lignin present can also affect the recalcitrance of the lignocellulosic feedstock. A higher ratio of syringyl to guaiacol subunits (*S*/*G* ratio) present in the lignin has previously been suggested to improve the saccharification yield of *P. trichocarpa* and subsequent ethanol yield from fermentation of the enzymatically saccharified biomass with yeast [32]. For the lines tested here with available data on S/G ratio, the higher ratios correlated weakly with increased fermentation performance (*R*^2^ = 0.41) (Fig. 3). The importance of the *S*/*G* ratio has been reported in prior work with various *Populus* species and ratios of such previous work are included in Table 1. While other work has highlighted the significance of the *S*/*G* ratio of lignin [5, 23, 32], no such a correlation of S/G to recalcitrance was noted with the microbial-based assay utilized in this study.

### Growth productivity of lignocellulosic feedstock

A viable lignocellulosic feedstock must not only be more readily digestible, either by a naturally cellulolytic and hemicellulolytic organism, such as *C. bescii*, or by more traditional enzymatic saccharification treatment, it must also have favorable growth performance and productivity. Lower lignin is generally correlated with growth defects [16]. Herein, we found a similar correlation with those lines demonstrating the highest fermentation performance, i20-5 and i35-7, having stem volumes of 50% and 26% of the wild type, respectively (Table 2). Yet, there are some transgenic lines in which the lower lignin content did not result in a growth defect. Line i20-10 (lignin content 13.3%), developed with the same construct as i20-5, targeting the C3H3 gene but with slightly less down-regulation, had a stem volume of 95% of the wild type, thus demonstrating low lignin composition and excellent fermentation performance without a penalty to biomass productivity.

**Fig. 4:**
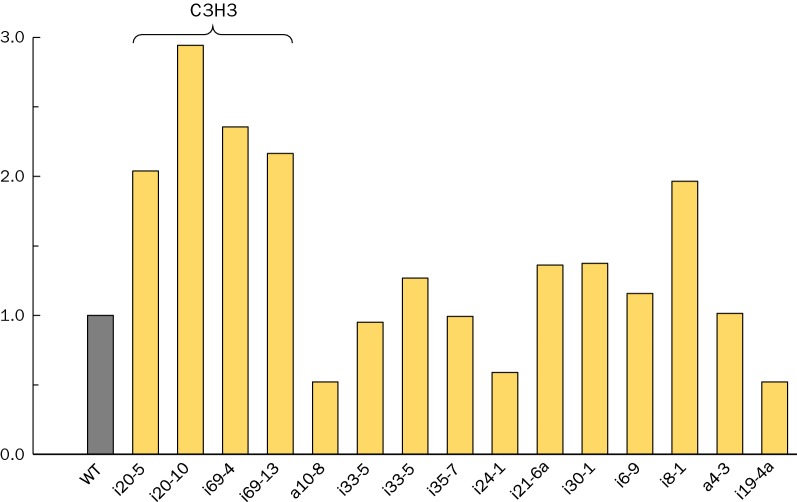
Fermentation-growth factor of transgenics compared to wild type for *C. bescii* fermentation. Fermentation-growth factor is the product of estimated stem volume (from [28] and fermentation product titer from *C. bescii* treatment). The wild-type control was set to 1.0 to normalize data. The four best performing lines (i20-5, i20-10, i69-4, and i69-13) all targeted the C3H3 gene. *Stem volume data not available for a12-10 and i15-3 such that fermentation-growth factor could not be calculated

To account for these parameters, a fermentation-growth factor was created in which the concentration of fermentation products (ethanol plus acetate, in mM) was multiplied by the stem volume (normalized to wild type) and the overall factor normalized to wild type set at 1.0 (Fig. 4). Transgenic poplar lines generated targeting the C3H3 gene stand out as lines with desirable properties for further improvement of biomass feedstocks. In fact, line i20-10 performs three times as well as the wild-type after accounting for both growth factors and fermentation performance. Thus, this suggests that the C3H3 gene is a highly promising target for low recalcitrance biomass. However, there may only be a narrow transcript window in which the growth phenotype is maintained for a less recalcitrant feedstock. The i20-5 line had a C3H3 transcript level of 13% and i20-10 was similar at 17%, both exhibiting greatly improved solubilization and fermentation with *C. bescii*. Line i20-2 also targeted the C3H3 gene and the C3H3 transcript level was approximately 50% of wild type. However, despite the transcript reduction to 50%, this line demonstrated lignin content, wood composition, and enzymatic saccharification results in line with wild type [28]. Thus, more control of transcript levels may be required to generate lignocellulosic feedstocks with the desired properties.

Many previous efforts to generate transgenic poplar, such as those listed in Table 1, have been performed by RNA interference using *Agrobacterium* based genetic techniques, which does not allow fine down-regulation control due to random genome integration of transgene. Thus, more surgical genetic tools are needed to exert precise control of transcript level, localization, and impact on specific cell types. CRISPR-based genome editing may be the solution to more strategic control of monolignol biosynthesis and, hence, the desired reduction in biomass recalcitrance.

## Conclusions

There have been attempts to reduce recalcitrance in various potential lignocellulosic feedstocks via other methodologies that do not involve the monolignol biosynthetic pathway. Examples include the overexpression of xylem development regulatory genes [8], down-regulation of pectin synthesis [1], and overexpression of cell wall degrading enzymes, such as xyloglucanases [17], glycosyl hydrolases [31] and xylanases [21]. Natural variants with desirable properties have also been considered [14, 23, 32]. However, these approaches have not yet achieved the reductions in feedstock recalcitrance obtained through the use of systems biology-based approaches for generating transgenic plants with strategic properties.

Utilization of a direct screen of lignocellulosic feedstocks, such as transgenic wood, by an organism capable of both deconstructing plant biomass and fermenting the carbohydrate content is an effective and informative alternative to assessment by enzymatic saccharification and fermentation. This is feasible utilizing lignocellulosic fermentative microbes, such as *C. bescii*, that not only digest and metabolize the hexose and pentose saccharide portions of unpretreated plant biomass but can themselves be genetically engineering to generate useful products. Consequently, as described herein, this microbial based assay provides insight into how genetic alterations to the transgenic plant affect biomass solubilization and conversion. While the fermentation capabilities of *C. bescii* require further improvement for consideration of this organism for commercial use, the utilization of such a screen provides another informative tool for characterizing biomasses proposed in a lignocellulosic feedstock bioprocess.

## Supplementary information

**Additional file 1.** Poplar biomass samples utilized in this study.

## Data Availability

The datasets generated or analyzed during this study are included in this published article and its additional file. Further data and materials are available upon reasonable request from the corresponding author.
