# Supplementary material for: Use of the lignocellulose-degrading bacterium Caldicellulosiruptor bescii to assess recalcitrance and conversion of wild-type and transgenic poplar
Source: Biotechnol Biofuels. 2020 Mar 11;13:43. doi: 10.1186/s13068-020-01675-2 (PMC7065347; doi:10.1186/s13068-020-01675-2)
Supplement: Supplementary file 1 — Additional file 1. Poplar biomass samples utilized in this study. [file 13068_2020_1675_MOESM1_ESM.docx]

| **Table S1.** Samples Utilized in this Study | | |
| --- | --- | --- |
|  | **Target Gene(s)** | **Samples from Wang et al** |
| i20-5 | C3H3 | i20-5-1 |
| i20-10 | C3H3 | i20-10-3 |
| i69-4 | C3H3 | i69-4-1 |
|  | C4H1 |  |
|  | C4H2 |  |
| i69-13 | C3H3 | i69-13-1 |
|  | C4H1 |  |
|  | C4H2 |  |
| a10-8 | C4H1 | a10-8-1 |
| a12-10^a^ | 4CL3 | a12-10-1, a12-10-3, a12-10-3 |
| i15-3^a^ | 4CL3 | i15-3-1, i15-3-2, i15-3-3 |
|  | 4CL5 |  |
| i33-5 | CAD1 | i33-5-1 |
| i33-5 | CAD1 | i33-5-2 |
| i35-7 | CAD1 | i35-7-1 |
|  | CAD2 |  |
| i24-1 | CCoAOMT1 | I24-1-2 |
|  | CCoAOMT2 |  |
| i21-6^a^ | CCoAOMT3 | i21-6-1, i21-6-2, i21-6-3 |
| i30-1 | AldOMT2 | i30-1-1 |
| i6-9 | PAL1 | i6-9-1 |
|  | PAL2 |  |
| i8-1 | PAL1 | i8-1-3 |
|  | PAL2 |  |
|  | PAL3 |  |
|  | PAL4 |  |
|  | PAL5 |  |
| a4-3 | PAL5 | a4-3-1 |
| i19-4^a^ | HCT1 | i19-4-1, i19-4-2, i19-4-3 |
|  | HCT6 |  |
| ^†^Poplar Samples obtained from (Wang et al., 2018)  ^a^Technical replicate tree samples were pooled to obtain sufficient material for testing. | | |
